# The interaction of gait characteristics and concerns about falling in community-dwelling older adults

**DOI:** 10.1038/s41598-025-32000-2

**Published:** 2025-12-12

**Authors:** Sabine Britting, Sebastian Krumpoch, Ulrich Lindemann, Anja Müller, Nicolas Rohleder, Toby Jack Ellmers, Alex Burkard, Ellen Freiberger, Robert Kob

**Affiliations:** 1 Department of Internal Medicine-Geriatrics, Institute for Biomedicine of Aging, Friedrich-Alexander-Universität Erlangen-Nürnberg, Kobergerstr. 60, 90408 Nürnberg, Germany; 2 Department of Geriatrics, Robert Bosch Hospital, Auerbachstraße 110, 70376 Stuttgart, Germany; 3 Department of Psychology, Chair of Health Psychology, Friedrich-Alexander-Universität Erlangen-Nürnberg, Nägelsbachstr. 49a, 91052 Erlangen, Germany; 4 Centre for Vestibular Neurology, Department of Brain Sciences, Imperial College London, London, UK

**Keywords:** Gait characteristics, Gait, Concerns about falling, Older adults, Falls, Engineering, Health care, Risk factors

## Abstract

Concerns about falling (CaF) are common in older adults and are associated with increased falls. Although cautious gait—a gait pattern linked to CaF—has been described, the specific gait parameters most strongly associated with CaF remain unclear. This study investigates the association between gait characteristics at normal and maximal gait speed and CaF in community-dwelling older adults. This cross-sectional analysis merged data from two studies including participants aged 65 years and older: the FEARFALL-study, an intervention study to reduce CaF and improving walking stability, and from the MOGA-study, investigating the harmonization of supervised short-walk test protocols. Gait analysis was performed using an instrumented walkway and CaF was assessed by the Falls Efficacy Scale International (FES-I). Multiple stepwise regression models were used to explore the association of gait parameters with CaF and the associations of individual FES-I items with gait speed. Data from 261 participants (MOGA-study n = 150; FEARFALL-study n = 111; mean age 80.0 (± 4.6) years; 67% women) were analysed. Increasing FES-I levels were associated with decreasing walking performance across most gait parameters. Normal walking speed explained 24.9% of the variance in FES-I increasing to 29.2% by adding maximum gait speed and walk ratio. Four FES-I items dedicated to dynamic balance could be used to screen for cautious gait (adjusted R^2^ = .201). Gait variables, especially gait speed, are strongly associated with CaF. FES-I items related to dynamic balance might be used to screen for cautious gait in community-dwelling older adults.

## Introduction

Falls are major issues for older adults resulting in significant physical impairment, morbidity and sometimes in death at the individual level^1,2^, and in additional costs at the community level^3^. Consequently, may older adults experience concerns (i.e. “fears”) about falling (CaF)^4^, defined as a “lasting feeling of dread and apprehension about situations that are believed to threaten or challenge balance”^5^. The prevalence of CaF ranges from 20–39% in older persons^6^ and increases to 40–73% in those who have fallen^7^. CaF is associated with various negative outcomes in community-dwelling older adults, including social isolation^8^ greater functional decline^9^ and increased risk of future falls^10^. CaF is commonly obtained by either a single question (yes/no) or the Falls Efficacy Scale International (FES-I)^11^. The FES-I has demonstrated excellent psychometric properties^12^. It is an in-depth assessment asking 16 questions covering different aspects of CaF, such as walking, dressing, or socializing. Delbaere et al.^13^ provided cut-off scores for low, moderate and high levels of CaF.

As most falls occur while walking^14,15^, most fall-risk assessments include a measure of walking performance (e.g., see the 2022 World Falls Guidelines)^1^. In clinical settings routinely only one aspect of walking performance, i.e. gait speed, is measured by stopwatch. For research questions, an in-depth assessment of walking performance can easily be performed by instrumented walkways^16^ or by body-worn sensors^17–19^ measuring different aspects of walking, such as walking capacity, symmetry, regularity, coordination, dynamic balance, foot movement^20–22^. A better walking stability, e.g. holding a therapist´s hand or a walker, suggesting lower CaF, slightly improves walking performance with faster gait speed and reduced gait variability^23^. Conversely, one study has shown an impaired walking performance after exposing older women to a fictional disturbing factor, suggesting increased CaF^24^. All these studies have focussed only on a limited number of gait parameters.

Research has demonstrated associations between CaF and gait, terming it “cautious gait”^25,26^. These studies have shown that CaF influences spatial and temporal gait parameter changes in older persons leading to slower gait speed, shorter stride length, increased stride width, and prolonged double limb support time. Importantly, these associations occur independently of fall history, with such behaviors similarly observed in non-fallers who nonetheless have CaF^27^. Overall, “cautious gait” seems to indicate an association between CaF and gait parameters independent of a fall history. However, a comprehensive gait analysis is time-consuming and needs special, costly equipment. Furthermore, not all parameters could be measured with every gait analysis system. Therefore, it is crucial to establish a core set of variables to identify cautious gait. Additionally, a pre-screening with the established FES-I questionnaire for potentially affected persons is useful to avoid unnecessary costly assessments.

To date, it is also unclear if cautious gait patterns are best measured at normal or maximal gait speed under laboratory conditions. Normal gait speed is a general marker of physical performance^22^. It is predictive for mortality^28^, hospitalization^29^, frailty^30^, and future falls^31^. Gait characteristics at maximum gait speed condition might better show underlying neuromuscular decline while its detection is limited in the normal gait speed condition^32^. In addition, maximum gait speed declines at a faster rate than normal gait speed^33^. Maximum gait speed is associated with muscle strength^34^ and is clinically relevant for frail older adults^35^, persons after stroke^36^, and persons with Multiple Sclerosis^37^.

Therefore, the first aim of the current manuscript was to identify a core set of gait characteristics to measure cautious gait easier in research as well as clinical settings. The second aim was to determine whether gait at normal speed, maximum speed, or both have to be recorded for assessment of cautious gait. The third aim was to identify specific items of the FES-I that might be used as red flags for the screening for cautious gait.

## Methods

### Subjects and design

The data of 2 studies were merged for this secondary cross-sectional analysis to enhance statistical power. From the FEARFALL-study, an intervention study to reduce CaF and to improve balance and lower extremity strength by physical exercise in community-dwelling older adults^38^, the baseline data were used. Furthermore, the data of the MOGA-study were included, a cross-sectional study to generate further knowledge for the harmonization of supervised short-walk test protocols in community-dwelling older adults^39^. Participants of both studies were recruited through leaflets in community senior meetings, pharmacies, medical practices and through the local press in the area of Nuremberg/Fuerth/Erlangen, Bavaria, Germany. Inclusion criteria for both studies were an age of 65 years or older and sufficient mobility to travel to the study centre. Specific inclusion criteria were a FES-I score of 20 or higher for the FEARFALL-study, and the ability to walk without a walker/wheeled walker for the MOGA-study. Exclusion criteria for both studies were orthopaedic and/or neurologic problems affecting walking performance (self-report) and any medical, psychiatric, or behavioural problems that, in the judgement of the principal investigator, could interfere with the ability to follow the study protocol. The FEARFALL and MOGA study protocols were approved by the ethical committee of the medical faculty of the Friedrich-Alexander-Universität Erlangen-Nürnberg (#317_20B) and (#43_19B), respectively. All participants of both studies gave written informed consent. All study procedures were performed in accordance with the guidelines published in the Declaration of Helsinki.

### Outcome assessments

All assessments were conducted in the gait laboratory of the Institute for Biomedicine of Aging (IBA) at Friedrich-Alexander-Universität Erlangen-Nürnberg. Gait analysis was performed in the well-lit laboratory without external distraction. Participants wore their own outdoor shoes which were closed around the heels and walked both at their normal pace and at their maximum pace over an 8-m-long instrumented walkway (GAITRite, CIR Systems, Franklin, USA) with additional 2.5 m each for acceleration and deceleration^40^. Two walks, each with normal and maximum pace, were performed and the second walk was used for analysis. Included parameters were all gait parameters reflecting pace, symmetry, regularity, spatiotemporal coordination, and dynamic balance. The walk ratio was calculated by diving the mean step length by the cadence. The step length differential is defined as the difference between the step length of the right and the left foot.

CaF was assessed by the FES-I^11^, a 16-item questionnaire asking for CaF related to different situations in daily-life. Each question was scaled from 1 (not concerned at all) to 4 (fully concerned), resulting in a 16 to 64 total score with higher scores representing higher CaF. The total score was used as primary outcome. The cut-offs proposed by Delbaere et al. for low (16–19), moderate (20–27) and high CaF (28–64) according to the FES-I were used^13^.

### Descriptive assessment

Anthropometric measures to describe the cohort were age, body height and body weight. General physical performance was described by the Short Physical Performance Battery (SPPB)^41^ including tests of walking, chair rise, and static balance. In addition, the Geriatric Depression Scale^42^ was used to obtain information on depressive symptoms. CaF and normal gait speed were also used as descriptive parameters.

### Statistics

All parameters were described by mean, standard deviation, minimum and maximum. Normality and outliers were identified by visual inspection of the Q-Q and box plots. Groups were either compared with one-way ANOVA for continuous variables or chi-squared tests for categorical variables. P values were adjusted according to Tukey to account for multiple testing. Associations between parameters were described by Spearman´s coefficients of correlation. Multiple stepwise regression models with p < 0.05 for inclusion and p > 0.05 for exclusion of variables were used to show the effect of in-depth gait parameters (independent parameters) on CaF (dependent parameter) and the effect of FES-I single items (independent parameters) on gait speed (dependent parameter). All models were adjusted for age, sex and body height. Multi-collinearity was checked by the variance inflation factor (VIF). Homogeneity of variances was verified using Levene’s Test. A most important feature analysis was performed based on p-values and a most affected parameter analysis was performed based on change of R^2^ values. All statistics were performed using IBM SPSS Statistics 30.0 (IBM Corp., Armonk, USA). P values < 0.05 were regarded as statistically significant.

## Results

Data sets of 261 community-dwelling older adults (MOGA-study n = 150; FEARFALL-study n = 111) with a mean age of 80.0 (± 4.6) years (67% women) were analysed. A detailed description of the cohort is shown in Table 1.

**Table 1:** Description of the total (n = 261), MOGA (n = 150), and FEARFALL (n = 111) cohort of community-dwelling older adults.

Variable	Total (n = 261) Mean ± SD [Range]	MOGA (n = 150) Mean ± SD	FEARFALL (n = 111) Mean ± SD	p
Gender (women/men) [n]	174/87	92/58	82/29	0.034
Age [years]	80.0 ± 4.6 [70–93]	80.5 ± 4.5	79.3 ± 4.8	0.043
Body height [cm]	165.8 ± 10.6 [141.5–197.5]	163.8 ± 10.4	168.6 ± 10.3	< 0.001
Body mass [kg]	74.9 ± 15.8 [43.9–121.9]	74.9 ± 16.2	74.8 ± 15.2	0.963
FES-I [score]	25.0 ± 8.1 [16–56]	21.2 ± 5.7	30.1 ± 8.3	< 0.001
SPPB [score]	10.1 ± 2.1 [2-12]	10.9 ± 1.7	9.1 ± 2.3	< 0.001
GDS [score]	1.9 ± 2.0 [0–11]	1.7 ± 2.0	2.2 ± 2.1	0.035
Normal gait speed [m/s]	1.2 ± 0.3 [0.3–1.8]	1.2 ± 0.3	1.1 ± 0.2	< 0.001
Maximal gait speed [m/s]	1.5 ± 0.3 [0.3–2.4]	1.5 ± 0.3	1.5 ± 0.3	0.074
Stride length, normal [cm]	123.1 ± 20.6 [56.5–173.0]	119.7 ± 19.1	125.7 ± 21.3	0.021
Stride length, maximal [cm]	137.7 ± 24.8 [53.7–177.5]	134.1 ± 23.2	140.3 ± 25.7	0.044
Stride length variability, normal [%]	3.2 ± 1.8 [0.8–13.3]	3.1 ± 1.7	3.3 ± 1.9	0.307
Stride length variability, maximal [%]	3.4 ± 1.9 [0.8–12.9]	3.5 ± 1.9	3.6 ± 1.9	0.843
Step length differential, normal [cm]	2.7 ± 2.5 [0.0–18.5]	2.8 ± 2.8	2.7 ± 2.0	0.820
Step length differential, maximal [cm]	3.0 ± 2.6 [0.0–14.3]	3.0 ± 2.7	3.1 ± 2.4	0.910
Step width, normal [cm]	9.4 ± 3.8 [1.9–24.5]	9.2 ± 3.8	9.7 ± 3.7	0.355
Step width, maximal [cm]	9.5 ± 3.8 [2.0–26.0]	9.5 ± 3.8	9.2 ± 3.7	0.975
Step width variability, normal [%]	27.4 ± 14.8 [5.0–81.9]	26.8 ± 14.2	28.2 ± 15.5	0.444
Step width variability, maximal [%]	28.5 ± 16.1 [3.0–88.5]	27.3 ± 14.5	30.0 ± 18.0	0.199
Walk ratio, normal [cm/(1/cm)]	0.552 ± 0.086 [0.300–0.780]	0.554 ± 0.088	0.550 ± 0.085	0.719
Walk ratio, maximal [cm/(1/cm)]	0.531 ± 0.096 [0.260–0.800]	0.540 ± 0.096	0.519 ± 0.096	0.083
Cadence, normal [1/min]	111.8 ± 11.1 [68.2–148.5]	113.7 ± 10.9	109.3 ± 11.0	0.002
Cadence, maximal [1/min]	130.4 ± 15.1 [65.4–186.2]	130.5 ± 15.1	130.2 ± 15.3	0.891
FES-I: Falls Efficacy Scale-International, GDS: Geriatric Depression Scale, SD: Standard deviation, SPPB: Short Physical Performance Battery; Differences between the two included studies with p < 0.05 were regarded as significant				

Different FES-I levels showed a clear pattern of decreasing walking performance in nearly all in-depth gait parameters with increasing CaF (Table 2).

**Table 2:** Description of the study cohort according to different FES-I levels.

		FES-I		
Variable	Low (16–19) (n = 76) Mean ± SD	Moderate (20–27) (n = 107) Mean ± SD	High (28–64) (n = 76) Mean ± SD	p
Gender (women/men) [n]	40/36	78/30	56/21	0.010
Age [years]	79.6 ± 4.1	79.9 ± 4.7	80.5 ± 5.1	0.503
Body height [cm]	166.3 ± 10.6	165.2 ± 10.8	166.3 ± 10.5	0.696
Body mass [kg]	73.6 ± 14.8	75.6 ± 16.6	75.4 ± 15.7	0.653
FES-I [score]	17.4 ± 1.1	23.0 ± 2.3*	35.3 ± 7.1^#,§^	< 0.001
SPPB [score]	11.5 ± 0.9	10.2 ± 1.9*	8.7 ± 2.5^#,§^	< 0.001
GDS [score]	1.0 ± 1.4	2.0 ± 1.9*	2.7 ± 2.4^#,§^	< 0.001
Normal gait speed [m/s]	1.3 ± 0.2	1.1 ± 0.2*	1.0 ± 0.2^#,§^	< 0.001
Maximal gait speed [m/s]	1.7 ± 0.2	1.5 ± 0.3*	1.3 ± 0.4^#,§^	< 0.001
Stride length, normal [cm]	135.6 ± 15.6	122.1 ± 18.3*	112.3 ± 21.6^#,§^	< 0.001
Stride length, maximal [cm]	150.9 ± 18.3	138.4 ± 23.7*	123.7 ± 24.8^#,§^	< 0.001
Stride length variability, normal [%]	2.5 ± 0.9	3.2 ± 1.8*	3.8 ± 2.2^#,§^	< 0.001
Stride length variability, maximal [%]	2.7 ± 1.4	3.3 ± 1.8	4.2 ± 2.2^#,§^	< 0.001
Step length differential, normal [cm]	2.6 ± 2.6	2.6 ± 2.6	3.0 ± 2.2	0.420
Step length differential, maximal [cm]	2.9 ± 2.8	2.8 ± 2.4	3.4 ± 2.5	0.321
Step width, normal [cm]	8.0 ± 2.4	9.4 ± 3.7	10.8 ± 4.5^#,§^	< 0.001
Step width, maximal [cm]	8.6 ± 2.9	9.3 ± 4.0	10.7 ± 4.0^#,§^	0.003
Step width variability, normal [%]	28.8 ± 13.8	27.5 ± 14.2	26.1 ± 16.6	0.560
Step width variability, maximal [%]	27.2 ± 13.1	30.5 ± 17.6	27.1 ± 16.7	0.268
Walk ratio, normal [cm/(1/cm)]	0.585 ± 0.077	0.547 ± 0.081*	0.526 ± 0.093^#^	< 0.001
Walk ratio, maximal [cm/(1/cm)]	0.568 ± 0.089	0.532 ± 0.092*	0.496 ± 0.096^#,§^	< 0.001
Cadence, normal [1/min]	116.6 ± 9.3	111.8 ± 10.2	106.9 ± 12.1^#,§^	< 0.001
Cadence, maximal [1/min]	134.1 ± 13.0	130.4 ± 12.8	126.0 ± 18.6^#,§^	0.008

FES-I: falls efficacy scale-international, GDS: geriatric depression scale, SD: standard deviation, SPPB: short physical performance battery; anova with post-hoc group comparisons * Significant difference between group 1 and 2; # Significant difference between group 1 and 3; § Significant difference between group 2 and 3; significance < 0.05 adjusted according to Tukey.

The associations between CaF and normal gait speed, and CaF and maximum gait speed were r = -0.477 (p < 0.001) and r = -0.454 (p < 0.001), respectively. A stepwise regression model with FES-I as dependent variable and gait in-depth parameters as independent variables was able to explain 29.2% of the variance of the FES-I (Table 3). Here, gait speed and walk-ratio at normal as well as maximum velocity were the in-depth parameters significantly adding to the explained variance in CaF.

**Table 3:** Stepwise regression model with FES-I as dependent variable and gait in-depth parameters as independent variables with all models adjusted for age, sex and body height.

	Corrected R^2^ of whole model	Change of R^2^ from last model	p for change of R^2^ from last model
1.: Normal gait speed	0.249	0.249	< 0.001
2.: 1 + walk ratio at maximal gait speed	0.266	0.017	0.010
3.: 2 + walk ratio at normal gait speed	0.280	0.014	0.014
4.: 3 + maximal gait speed	0.292	0.012	0.024

As the addition of further in-depth gait parameters to normal gait speed showed only minor changes in explained variance with CaF, the further analyses were only carried out with normal gait speed. A stepwise regression model with normal gait speed as dependent variable and all FES-I in-depth parameters as independent variables was able to explain 20.1% of the variance of the FES-I (Table 4). Here, 75% of the most important parameters were related to walking (walking on uneven ground, going up or down stairs, walking around in the neighborhood) whilst the remaining items related to dynamic balance more generally (cleaning the house).

**Table 4:** Stepwise regression model with normal gait speed as dependent variable and all FES-I in-depth parameters as independent variables with all models adjusted for age, sex and body height.

	Corrected R^2^ of whole model	Change of R^2^ from last model	p for change of R^2^ from last model
1.: Walking on uneven ground	0.136	0.136	< 0.001
2.: 1 + Going up or down stairs	0.166	0.030	< 0.001
3.: 2 + Cleaning the house	0.188	0.022	0.004
4.: 3 + Walking around in the neighbourhood	0.201	0.013	0.022

## Discussion

In this study we investigated the association between concerns about falling (CaF) and gait characteristics. Previous studies had reported inconsistent results regarding which specific gait variables are most strongly associated to CaF in community-dwelling older persons. In some studies, higher level of concerns led to changes in spatial and temporal gait characteristics^25^, whereas in other studies, gait unsteadiness was closely associated with CaF^43^. Overall, we found that most gait speed variables differed significantly between the different levels of CaF.

Our participants were recruited from two different studies showing differences in the level of CaF, as well as functional status, measured by the SPPB. Regarding gait differences, only normal gait speed and cadence, both assessed at normal gait speed were significantly different between the two cohorts. No other relevant differences e.g. age, sex distribution or other gait characteristics were found, supporting the homogenous sample for our investigation.

In the descriptive analyses, we found significant differences for stride length, step width, and walk ratio depending on the level of CaF indicating a cautious gait pattern in our participants. Our results are consistent with those of other studies^25,26^. In particular, the significant association between normal gait speed and CaF has also been identified by other researchers^25,44^. However, the step length differential was not associated with CaF in our sample. This may be because this parameter is mostly altered by diseases that have a high impact on walking performance, such as stroke or Parkinson´s disease^45,46^. Lindemann^22^ assigns this parameter to the category of gait symmetry, which is primarily disease oriented. This indicates that the health status of the subjects has an influence on the significance of the parameters to be examined^22^. These conditions were excluded from our studies and the remaining variance in our community-dwelling population may have been too small to reveal an association with CaF. Our regression analyses of normal gait speed explained ~ 25% of the variation in CaF levels by considering only normal gait speed. Therefore, a simple analysis of normal gait speed, which only requires a 4-m walking section and a stopwatch, could be used to identify cautious gait. Interestingly, combining normal and maximum walking speed with the walk ratio could additionally explain about 4% of the variation in the CaF levels, leading to an overall explanation of 30%. However, as the walk ratio is based on cadence and step length, further technical equipment would be necessary to obtain these in-depths gait characteristics to investigate their association with CaF.

Interestingly, especially the questions regarding dynamic balance in the FES-I “walking on uneven ground”, “going up and down the stairs” and “walking around the neighbourhood” together explained 20% of the variance of normal gait speed between the participants. Therefore, higher reported CaF within these questions could be a sign for a cautious gait pattern that should be assessed by further measurements of gait characteristics. The questions regarding more static balance e.g. “preparing a meal” or “reaching something above your head” were not associated with gait variables. This underlines former findings that in challenging situations the level of concerns is increasing. This was especially shown in postural control studies in which fear of falling was induced, by raising the height (or perceived height) of a support platform^47^.

Research has shown that cautious gait can be categorized as higher level gait disorder when it cannot be explained by deficits in strength, tone, sensation, or coordination but rather to neuropsychological factors^48^. This approach includes a conscious process in monitoring and controlling movements to reduce the risk of falling. For example, consciously movements controlling is less automatic, needs a longer initiation and greater cognitive resources, and the actual movements are slower and less efficient^48^. Our data supports this approach that CaF is having a significant impact on specific gait characteristics with regard to gait speed, stride length variability, step width, step width variability, walk ratio and cadence of the normal as well as the maximum gait speed walk.

In our study, including healthy older community-dwelling persons the gait variable step length differential could not show any impact as this variable is more relevant in stroke or hip fracture older patients. Therefore, our results cannot be transferred into other settings e.g. nursing home or hospital settings. In addition, we did not include fall history or other disease specific aspects. A further limitation of the current study was that we did not control for muscle strength or proprioception that might have influenced the measurements. Besides, the exclusion of persons with diseases that greatly alter gait performance limit the transferability of our results to the general population. However, there is a strong association between fall history, physical capacity limitations, CaF and future falls^10^. For this reason, the World Falls Guidelines recommend recording CaF, as this increases the risk of avoiding mobility and falls^1^. However, to date less is known about which gait characteristics are associated with CaF and might be part of this downhill spiral. Therefore, the cross-sectional design of the current study is not able to resolve this chicken-and-egg problem but could identify gait parameters that might moderate this process. Additionally, the question remains open, whether gait symmetry has a higher significance in the clinical setting. Taken together, further research is needed to investigate the association between gait variables and CaF levels in a broader population.

A strength of our study is the in-depths gait pattern and variables and their association with different CaF levels in community-dwelling older persons. This is to the authors understanding the first study relating gait speed, and gait patterns to the different categorization of CaF in community-dwelling older persons. Thus, providing further information in the area of fall prevention.

In conclusion, the present findings highlight a clear association between CaF and cautious gait behavior. Dynamic balance-related items of the FES-I demonstrated the highest impact on gait, and might be further investigated as early markers of gait changes. This cautious gait pattern could be analyzed by combined assessment of normal as well as maximum gait speed and walk ratio.

## Data Availability

The datasets generated during this study are not publicly available, but are available from the corresponding author on reasonable request.
